# Complete Genome Sequence of the Uropathogenic *Escherichia coli* Strain NU14

**DOI:** 10.1128/genomeA.00306-17

**Published:** 2017-05-04

**Authors:** Kurosh S. Mehershahi, Swaine L. Chen

**Affiliations:** a National University of Singapore, Singapore; b Genome Institute of Singapore, Singapore

## Abstract

*Escherichia coli* is the most common bacterium causing urinary tract infections in humans. We report here the complete genome sequence of the uropathogenic *Escherichia coli* strain NU14, a clinical pyelonephritis isolate used for studying pathogenesis.

## GENOME ANNOUNCEMENT

Urinary tract infections (UTIs) are common infections of humans that primarily affect women. The majority of UTIs are caused by *Escherichia coli*, hence the term uropathogenic *E. coli* (UPEC). While antibiotic therapy is generally successful, UTI recurs in 24 to 44% of patients within 6 to 12 months, often with a strain identical to that causing the initial infection (1). A fraction (2 to 5%) of patients further suffer from frequent recurrences (≥3 per year) (2). In a mouse model of UTI (3, 4), human UTI isolates not only infect the lumen of the bladder but also form intracellular collections within the epithelial cells of the bladder, which are known as intracellular bacterial communities (IBCs) and quiescent intracellular reservoirs (QIRs) (5, 6). IBCs and QIRs contribute to phenotypic antibiotic resistance, host immune evasion, and recurrent infection (7). Furthermore, IBC-like structures have been identified in the urine of adult and pediatric UTI patients (8–10). Thus, intracellular infection is a leading hypothesis for why humans suffer from recurrent UTI. The UPEC strain NU14 was isolated from a UTI patient (11). It was the first strain to be observed infecting epithelial cells in a mouse UTI model (12), and it was among several strains initially demonstrated to form IBCs (6). The initial genetic identification of the type 1 pilus adhesin (FimH) was also performed for NU14 (13), which led to its use in vaccine studies for UTIs (14).

NU14 genomic DNA was sheared to a size of approximately 10 kb using a g-Tube (Covaris). A SMRTbell library was prepared according to manufacturer’s instructions, loaded with a MagBead-bound library protocol, and sequenced on three SMRTCells using P5-C3 chemistry on the PacBio RSII instrument (Pacific Biosciences) with a 180-min movie time. *De novo* assembly was performed with the Hierarchical Genome Assembly Process (HGAP2) in the SMRT Analysis suite version 2.3 using all default parameters, except that the assembly target coverage parameter was changed to 20 (15). In addition, inspection of the assembled sequence and of the results from the BridgeMapper protocol led to manual resolution of an inverted sequence of ~315 bp, which corresponded to the invertible *fimS* promoter element. This was set to the “OFF” orientation, corresponding to the orientation in most other *E. coli* genomes. The sequence was then polished with data from the same SMRTCells using the Resequencing protocol in the SMRT Analysis suite. In total, there were 162,908 reads and 1,607,174,902 nucleotides that passed filtering, representing an approximate coverage of 300× (based on the final assembly) and a preassembly mean read length of 9,865 bp.

The NU14 genome consists of a single circular chromosome of 5,076,615 bp with a G+C content of 50.6%. Annotation of the NU14 genome was performed using the NCBI Prokaryotic Genome Annotation Pipeline (PGAP) (16). The NU14 chromosome contains 4,908 protein-coding sequences, 22 rRNAs, and 89 tRNAs. The finished genome sequence of UPEC strain NU14 will facilitate genetic manipulation and dissection of the virulence mechanisms of UPEC.

### Accession number(s).

The complete sequence of the UPEC strain NU14 chromosome has been submitted to GenBank under the accession number CP019777 (BioProject PRJNA373796).
